# Tokenization of social media engagements increases the sharing of false (and other) news but penalization moderates it

**DOI:** 10.1038/s41598-023-40716-2

**Published:** 2023-08-22

**Authors:** Meysam Alizadeh, Emma Hoes, Fabrizio Gilardi

**Affiliations:** Department of Political Science, University of Zurich, Zurich, Switzerland

**Keywords:** Human behaviour, Information technology

## Abstract

Some major social media companies are announcing plans to tokenize user engagements, derived from blockchain-based decentralized social media. This would bring financial and reputational incentives for engagement, which might lead users to post more objectionable content. Previous research showed that financial or reputational incentives for accuracy decrease the willingness to share misinformation. However, it is unclear to what extent such outcome would change if engagements instead of accuracy were incentivized, which is a more realistic scenario. To address this question, we conducted a survey experiment to examine the effects of hypothetical token incentives. We find that a simple nudge about the possibility of earning token-based points for the achieved user engagements increases the willingness to share different kinds of news, including misinformation. The presence of penalties for objectionable posts diminishes the positive effect of tokenization rewards on misinformation sharing, but it does not eliminate it. These results have policy implications for content moderation practices if platforms embrace decentralization and engagement tokenization.

## Introduction

Many decentralized social media (DeSo) platforms are offering various incentives to users for their activities on the platform. A DeSo is a platform that does not have a central governing body and operates on a blockchain^1^. Usually, users are rewarded with cryptocurrencies, commonly called token or coin (see Appendix S1 for background), for their (1) content creation, (2) content curation, (3) content attention, and (4) community management^2^. Following^3^, we define platform-issued tokens as “as an asset that conveys a right to the services of the platform and possible participation in its governance, but not necessarily cash flow rights”. For example, *Steemit* is a DeSo, analogous to Reddit, which has a price tag for each post that represents ‘Steem tokens’ that creators would earn for their achieved user engagement (e.g. up-vote or comment), encouraging them to post quality content^4^. The Steem token can then be exchanged for fiat money or other cryptocurrencies in supported online markets.

Major social media companies are announcing plans to incorporate elements of DeSo. Reddit introduced ‘Community Points’, which are a measure of reputation to reward users for their activity and quality content (see Appendix S3 for references on this and other similar plans of the major platforms). The points are on the blockchain, which means they can be converted to cryptocurrencies. In the beta version, for which users are invited to join the waitlist, users can only spend the ‘Reddit Coin’ on the platform. This includes tipping other users for their posts or comments, purchase special memberships, conduct large-scale polls, or reward developers for making tools for a subreddit. However, there are speculations about Reddit’s plan to eventually turn the coins to an Ethereum-based token, which would enable the owners to spend it anywhere (https://cointelegraph.com/news/reddit-to-reportedly-tokenize-karma-points-and-onboard-500m-new-users). Twitter introduced ‘Tips’, which allows users to support content by sending fiat money, Bitcoin, or Ethereum to creators. More recently, on the 5th of November 2022, Elon Musk announced in a tweet that “creator monetization for all forms of content” will soon be added to Twitter (https://www.businessinsider.com/elon-musk-twitter-monetization-model-forms-content-youtube-2022-11).

While the idea of DeSo is not new (see^1^ for a review of existing DeSo and Appendix S2 for a list of major existing DeSo platforms), the possibility of major platforms embracing tokenization incentives of DeSo is both promising and worrisome. On the one hand, it might help to alleviate problems linked to content moderation, (e.g., algorithmic ranking and managing objectionable content), data protection, advertisement pricing, and compensating content developers^2,3^. On the other hand, it may increase the spread of polarizing content^5^ and misinformation^6^ on social media if users are rewarded financially for their achieved user engagements. However, it may also decrease it if financial or reputational penalties are put in place^7^.

Even if major platforms do not incorporate these tokenization features in a near future, DeSo users are increasingly growing^1,8^ even though they are not necessarily familiar with all their features (see Appendix S10), which highlights the necessity of studying the consequence of token incentives. For example, after the recent purchase of Twitter by Elon Musk, the users of Mastodon, a decentralized social media platform similar to Twitter, increased by 100,000 in just a few days^9^. In addition, the financial mechanisms of DeSo are susceptible to misuse by free-riders who just promote others’ content^10^ or by bots^11^. In fact, a recent study showed that more than 16% of transfers of cryptocurrency in Steemit are sent to bots^4^.

**Figure 1:**
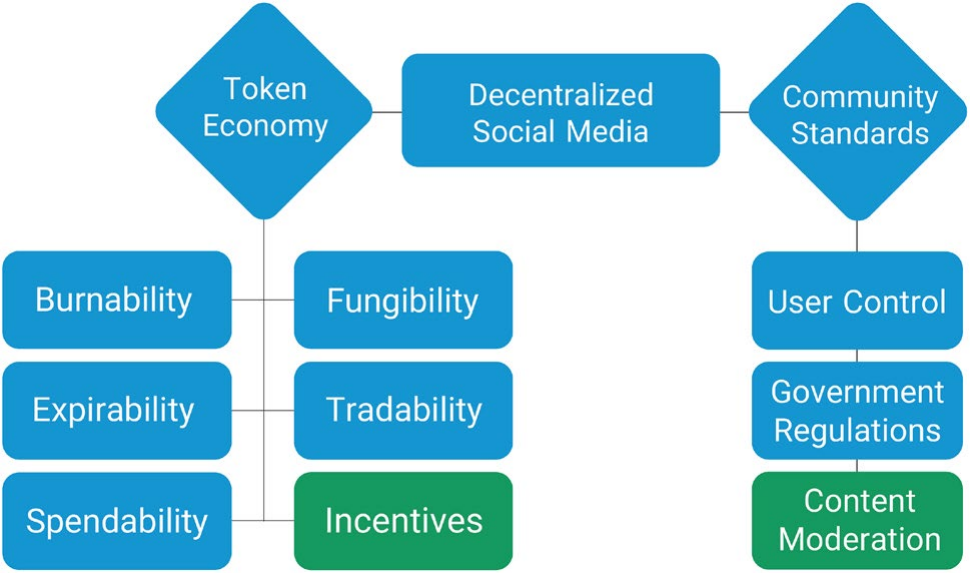
Main features of a decentralized social media that may affect the posting activity of users. Green boxes denote the parts studied in this paper. Burnability indicates whether a token can burned to terminate a right or revoke access. Expirability refers to whether a token can be expired after some time. Spendability indicates whether a token can be used to gain access to services or pay fees. Fungibility says if a token is interchangeable with other tokens. Tradability illustrates if a token can change ownership within a platform or on secondary markets. Finally, incentives schemes can be divided to two categories: (1) incentive enablers, which refer to what token holder can do with the token, and (2) incentive drivers, that indicate why a token holder engages with incentivized behaviour (e.g. gain reward or reputation)

To better understand how DeSo may affect the posting behavior of users, we put forward the framework of DeSo features in Fig. 1. “Token economy” is the redistribution of financial benefits to users through a platform-issued token^3^, and community standards includes users control over their content and profile, content moderation procedure, and government regulations. Tokens have many features that make them different from fiat money and more similar to stocks (see^12^ for a taxonomy). While all token features can potentially affect user behavior and require research, we argue that at the current stage of technology and public literacy, six features are the key that may affect the posting behavior of users (Fig. 1): (1) *Burnability*, indicating whether a token can burned to terminate a right or revoke access; (2) *Expirability*, referring to whether a token can be expired after some time; (3) Spendability, indicating whether a token can be used to gain access to services or pay fees; (4) *Fungibility*, referring to whether a token is interchangeable with other tokens; (5) *Tradability*, illustrating if a token can change ownership within a platform or on secondary markets; and (6) *Incentives*, referring to what token holder can do with it or why a token holder engages with incentivized behaviour (e.g. financial or reputational).

In this paper, our focus is on those DeSo features that are most likely to create problematic motivations: token incentives and content moderation regulations (Fig. 1). In our treatment, we assume that tokens do not burn or expire, are fungible, tradable, spendable, and, similar to Reddit’s community points, contain both financial and reputational incentives. More particularly, we are interested in studying the effect of rewarding and penalizing social media users with tokens on their willingness to share misinformation as well as neutral and hyperpartisan news headlines. As for content moderation, we consider a specific case that users would lose a portion of their token-based points should they post misinformation or hate speech. In short, we thus examine a situation in which users get token-based points for their achieved user engagements, and further explore under which circumstances their posting behavior changes.

The extant literature on the determinants of misinformation sharing intentions exhibits mixed results for our scenario. On the one hand, recent research demonstrates that accuracy nudging^13^, financial incentives for accuracy^14,15^ and reputational concerns^16^ all decrease the probability of sharing misinformation. Given that DeSo’s tokenization speaks to both financial and reputational motivations, it is thus to be expected that the implementation of engagement tokenization on social media platforms would make people less likely to share falsehoods. On the other hand, prior research shows that misinformation news providers receive higher median engagement than non-misinformation^17^, false news spread faster, farther, deeper, and more broadly than true news on Twitter^18^, political out-group posts are shared about twice as much as as in-group posts on Facebook and Twitter^19^, and partisan news is shared more frequently on Twitter^20^. Following this logic, it is to be expected that the implementation of engagement tokenization on social media platforms would make people more likely to share misinformation and polarizing news. These conflicting results motivate the need to investigate the extent to which tokenzation of social media engagements affect the users’ willingness to share different kinds of content, including misinformation but also other types of problematic content such as hyperpartisan news [A1] .

With respect to the content moderation aspect of our experiment (Fig. 1), a survey by Twitter showed that more than 90% of an international sample supported removing misleading and altered content when it was clearly intended to cause certain types of harm, and more than 75% believed that accounts sharing false and misleading information should be punished^21^. A more recent study found that despite significant differences along political lines, most Americans preferred quashing harmful misinformation over protecting free speech^22^. Building on a previous study on the effect of penalization on the spread of misinformation^23^, and consistent with our pre-registered hypothesis, it is to be expected that penalizing DeSo users by losing a portion of their token-based points for posting misinformation would decrease their willingness to share misinformation. However, the extent to which it diminishes the potential positive effect of engagement tokenization on misinformation sharing intention is unexplored.

In summary, our focus on tokenization of social media differs from previous research in five ways: (1) previous research studied incentives for being accurate^14^, whereas we study incentives for the obtained user engagements; (2) prior research studied the effect of financial *or* reputational incentives separately^14,16^, whereas we test the simultaneous effect of both through platform-issued tokens. In our scenario, the financial incentive are derived from the nature of token-based cryptocurrencies, and the reputational incentive are derived from the platform design. For example, Reddit explicitly defines its ‘community points’ as a measure of reputation which are displayed next to the usernames; (3) previous research on financial incentives involved receiving a fixed-value money^14^, whereas cryptocurrencies’ value might change in future, which brings *risk* (spend or convert to fiat money or keep for potential value increase) as another dimension into users’ decision making strategies; (4) prior research tested the effect of penalization without the existence of incentives^23^, whereas we test for joint effect of them and whether one can outplay the other; and (5) previous studies used hypothetical scenarios aiming at suggestions for improving major social media platforms, whereas ours is close to a near-future reality of them. It is important to note that our approach, which combines reputational and financial incentives, cannot determine the individual impact of each type of incentive. However, separating reputational and financial incentives would not align with the goals of our research, as the integration of these two aspects is central to the implementation of tokenization within DeSo.

As an increasing number of social media platforms are on the brink of incorporating tokenization incentives, insights about their potential negative consequences is urgently required for two reasons. First, inaction could exacerbate the spread of misinformation and its costs^24^ such as reduction in vaccination intent^25^. Second, there are currently some DeSo applications that have incorporated such incentives^1^. To this end, we propose a framework to understand how DeSo may affect user behavior and conduct an online survey experiment to examine how the tokenization incentives of DeSo might affect the likelihood of posting different kinds of content. We find that tokenization of the achieved user engagements increases the sharing of misinformation (as well as other kinds of news), which penalization compensates only partially. These results have policy implications for designing effective intervention strategies to reduce the spread of misinformation^24^ and preventing potential misuse of financial and reputational features by state-sponsored coordinated networks on social media^26^.

## Results

To provide evidence on the possible effects of the tokenization incentives of DeSo on users’ willingness to share different kinds of content, we conducted an online survey experiment with $n = 1501$ participants from a nationally representative U.S. sample (matched on sex, age, and ethnicity based on census data) recruited from Prolific. The survey was conducted between June 9, 2022, and June 12, 2022. In total, 1568 participants started the survey and 1501 of them completed it. The participants were presented with five true neutral, five misinformation, and five true hyper-partisan news headlines about COVID19. Following^27^, the headlines were randomly selected from the dataset in^28^ (see  “Materials and Methods” section). Participants were then asked about their willingness to share each of fifteen headlines on social media, which is our outcome variable. Participants in the treatment group were shown a set of statements about a hypothetical (but realistic) world in which major platforms reward their users with digital asset for their so-called *reputation points* (such as the Karma point in Reddit), calculated based on their achieved user engagements. While in all treatment conditions participants earn a (hypothetical) fixed reward, the treatments vary across two dimensions: (a) whether users could be penalized for posting misinformation or hate speech, and (b) whether the calculation of reputation points is explicit (see “Materials and Methods” section). The experimental design, data collection, and data analysis plan were preregistered (see “Materials and Methods” section).

**Figure 2:**
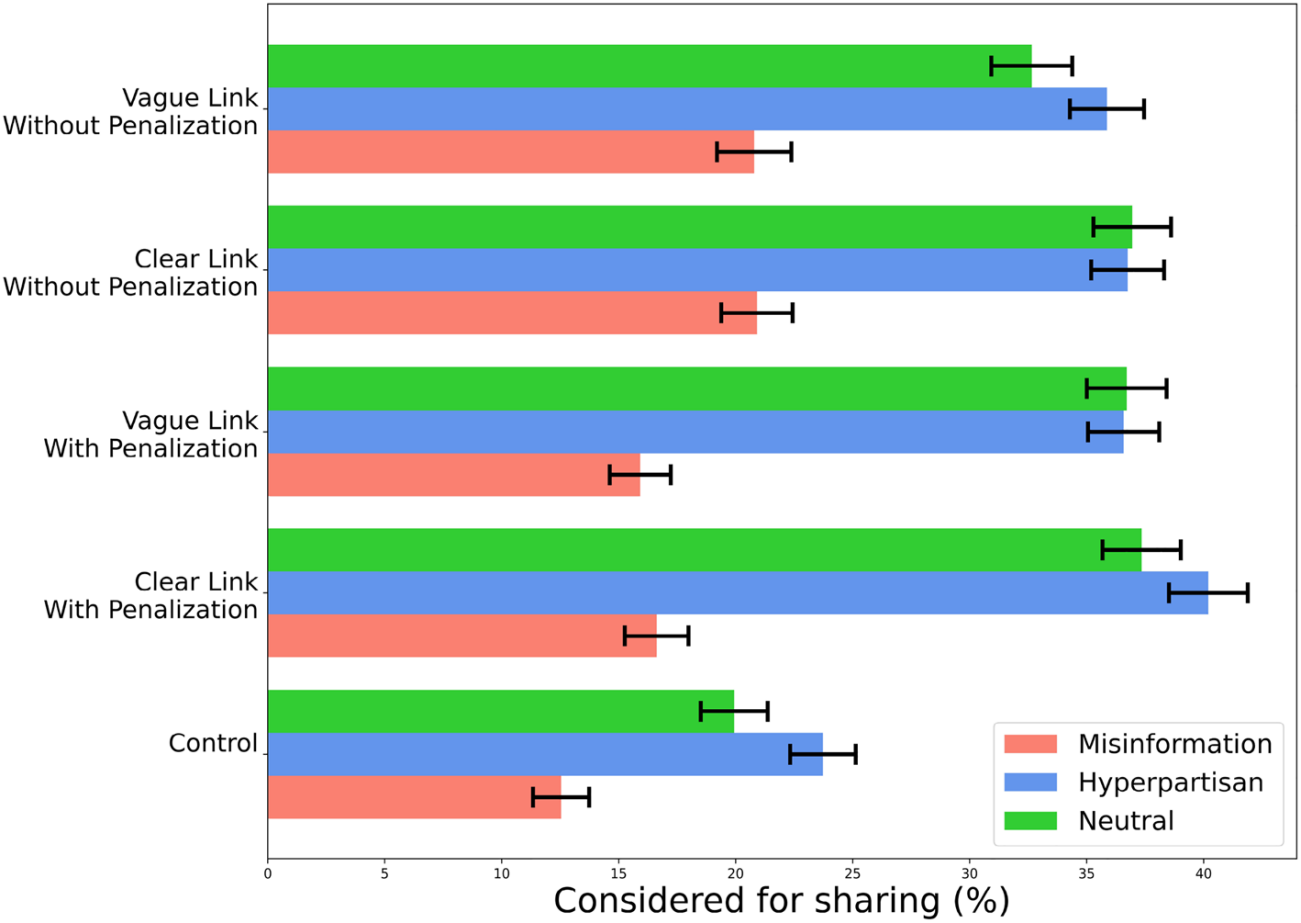
Fraction of various headlines that participants across the control and four treatment groups said they would consider sharing. In study 2, n = 1520 American individuals from Prolific were presented with a set of 15 headlines and asked to indicate whether they would consider sharing them on social media. Compared to control group, participants in all treatment conditions show higher sharing intention for all three types of headlines including misinformation and hyperpartisan news. Penalization would decrease the sharing intention of misinformation headlines but not to the level of the control group. Participants in the ‘clear link, with penalization’ condition show higher sharing intention of hyperpartisan news. Error bars indicate standard errors

To gain initial insights into whether tokenization incentives of DeSo impacts participants willingness to share various types of news headlines, we plot the fraction of headlines that participants across the control and treatments groups said they would consider sharing in Fig. 2. Compared to the control group, participants in all four treatment groups show higher sharing intention across all three types of news (12.8 percentage point difference on average). This includes 6.3 percentage point higher sharing intention of misinformation, 13.95 percentage point higher sharing intention of hyperpartisan, and 15.98 percentage point higher sharing intention of neutral news headlines. Participants in the ‘penalization’ condition show less sharing intention of misinformation compared to those in the ‘without penalization’ treatment condition (4.81 percentage point difference). However, penalization does not completely eliminate the the positive effect of token rewards. Participants in the ‘penalization’ condition show higher sharing intention of misinformation compared to the control group (3.89 percentage point difference). Whether the calculation of reputation points was made explicit or not does not seem to have any effect on the sharing intention of participants.

**Figure 3:**
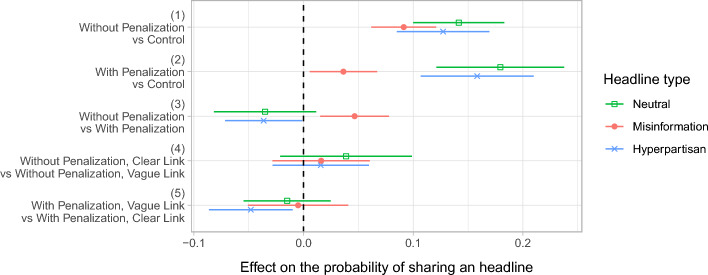
Point estimates from OLS regressions with 95% CI. Effect of token rewards for user engagements and penalties for problematic sharing behavior, as well as of the clarity of the link between rewards/penalties and user engagements/behavior, on the willingness to share neutral and hyperpartisan news, and misinformation. All treatment conditions include rewards. Without penalties, rewards increase the sharing intention of misinformation by about nine percentage points, compared to the control group. With penalties, the effect decreases to about four percentage points. The clarity of the link between rewards/penalties and user engagements/behavior has no effect

As per our preregistered analysis plan, Fig. 3 shows the effects of hypothetical token rewards, penalties, and calculation methods of reputation points (clear vs. vague) on the reported willingness to share neutral, hyperpartisan, and misinformation news headlines about COVID-19 on social media. The figure compares different scenarios: (1) rewards but no penalties, relative to the control group (i.e., neither rewards nor penalties); (2) both rewards and penalties, relative to the control group; (3) rewards but no penalties, relative to both rewards and penalties; (4) clear link, relative to vague link, between rewards and user engagements (no penalties in both scenarios); and (5) vague link, relative to clear link, between rewards and user engagements as well as penalties and posting behavior.

Taken together, we find that token rewards for user engagements increase the willingness to share all kinds of news including misinformation, and that token penalties for such behavior decrease the positive effect of rewards on the sharing of misinformation, but they do not eliminate it. More specifically, we highlight four sets of results. First, in the absence of penalties, token rewards for user engagements increase the willingness to share all kinds of news, including misinformation. Respondents are about nine percentage points more likely to share misinformation when they are rewarded with tokens for the user engagements their posts receive, relative to respondents for which no such rewards exist ($coef = 0.091, se = 0.015, p = 0.000$). Second, the same effect holds if respondents can be penalized for sharing misinformation, although effect size decreases to about four percentage points ($coef = 0.036, se = 0.016, p = 0.021$). Third, the effect of penalties is confirmed when comparing respondents within the treatment group, where rewards are in place. Given the presence of rewards, respondents that cannot be penalized for sharing misinformation are about five percentage points more likely to share misinformation than those who can be subject to penalties ($coef = 0.047, se = 0.016, p = 0.004$). Fourth, it does not matter whether respondents are told how exactly rewards and penalties are calculated. The clarity of the link between rewards and user engagements, and between penalties and sharing behavior, has no effect on the willingness to share content of any kind. As a robustness test, we re-estimated all models excluding those respondents who failed the manipulation check (see Fig S.2 in Appendix). The results are unchanged, with the exception of the effect of rewards and penalties relative to the control group. The point estimate remains positive but is smaller and not statistically significant ($coef = 0.021, se = 0.016, p = 0.185$).

## Discussion

The decentralization of social media will introduce a new set of incentives for users, which may affect their online behavior in both positive and negative ways. Given the existence of decentralized social media platforms and their increasing growth, and that most major social media platforms have announced plans in this direction, there has been speculation but little evidence regarding possible outcomes. Our approach combines reputational and financial incentives without explicitly isolating their individual impacts, which aligns with the integrated nature of tokenization in DeSo. The results, based on an online survey experiment, are of course limited (see^29^ for a discussion), but put forward evidence on the possible consequences of social media decentralization.

Our main conclusion is that rewarding users with tokens (i.e. a platform-issued digital asset such as cryptocurrency) for their achieved user engagements increase the news sharing intention of them including misinformation, hyperpartisan, and neutral news. However, if token penalties are set in place (i.e. losing tokens), that would disincentivize sharing intention of misinformation. Moreover, we find that rewards increase the willingness to share misinformation even when penalties are in place.

Since we have not asked participants about whether they think the news headlines are true or false, and what is the reason they are willing to share them, we are not able to statistically test for the potential mechanism(s) that could explain our findings. However, our results show that incentivizing user engagements increases the overall sharing intention of participants, which indicates our treatment simply make participants to share more of every types of news because every like, comment, or share matters for obtaining more community points. On the other hand, following previous research that show people think misinformation and polarizing content are more likely to go viral^30^, one might expects that participants already knew a news headline is false or polarizing and still decided to share with the hope of receiving more engagements. Therefore, future research should investigate the extent to which these mechanisms could explain the increase in sharing intention of people when engagements is incentivized. Furthermore, future research could study tokenization making different assumptions regarding the specific bundle of incentives involved and using different methods, including mock social media environments that can increase the realism of incentives. These steps would contribute to improving the generalizability of the findings.

These findings have policy implications for content moderation efforts^31^ especially that of curbing the spread of misinformation^32^, coordinated information operations^26^, conspiracy theories^33^, and bot activities^4^. More particularly, our findings that (1) token incentive outweighs token penalization, and (2) participants show higher sharing intention of hyperpartisan news when misinformation and hate speech are penalized are in agreement with the idea that people engage disproportionately higher with ‘borderline content’ including more sensationalist and provocative content^7^. The results further help to identify relevant directions for further research as well as potential issues that social media companies may need to consider as they incorporate elements of DeSo into their platforms. The decentralization of social media may further increase the misinformation problems that social media already face.

## Materials and methods

The study was approved by the Ethics Committee of the Faculty of Arts and Social Sciences, University of Zurich, on February 3, 2022. The experimental design, data collection, and data analysis plan were preregistered and carried out in accordance with relevant guidelines and regulations. We provided all participants with an informed consent form indicating their rights, that participation is voluntary, and they could drop out without any consequences. At the end of the experiment, all participants were presented with a debriefing statement (see Section S8 in Appendix) about the goal of the study and names and contact information of the researchers and the University of Zurich’s Ethics Committee. Preregistration materials are available at the Open Science Foundation (OSF). In all survey experiments, we do not exclude participants for inattentiveness or straightlining to avoid selection effects that can undermine causal inference.

### Participants

We preregistered a target sample of 1500 participants based on a power analysis aiming to obtain 0.80 power to detect a small effect size of 0.02 at the standard 0.05 alpha error probability. We also used a Bonferroni adjustment by dividing the nominal alpha level, 0.05, by the number of models tested, 15 (5 hypotheses, 3 outcomes), yielding an alpha of 0.003. The adjusted alpha level was used in the power analysis. The analysis revealed that we needed to recruit at least 730 participants (see Fig. S.1 in Appendix S5). We obtained a sample of nationally representative U.S. respondents (matched on sex, age, and ethnicity based on census data) recruited from Prolific. In total, 1569 started the survey and 1520 finished it. Only 98 participants indicated they have no social media account. However, we did not remove them from the dataset. We made this decision because one might expect that those without any social media account would join one in future if they know they can make money out of it. A further 459 participants (30%) did not pass the manipulation check (see  “Manipulation Check” section), but we did not remove them from the dataset. However, we re-run the analyses on only those who pass the manipulation check and report the results in Appendix S9 as a robustness check. The focused sample (mean age = 44.89) included 778 females, 727 males, and 15 other gender. This study was run on 10–12 June 2022.

### Materials

The participants were presented with 5 false, 5 true, and 5 true hyper-partisan news headlines about COVID19 in random order for each participant. All three types of news were randomly selected from a politically-balanced pool of news headlines. Following^27^, we selected all news headlines from^34^, which labeled news headlines in terms of accuracy and partisan orientation in the US. Participants in the treatment groups were presented with a set of statements about a hypothetical world in which major social media platforms decided to incorporate the tokenization incentives of decentralized social media platforms and reward their users with a digital asset for their so called *reputation points*, that is, being calculated in an explicit or implicit way based on the user engagements they received (i.e. like, share, comment, and video views). The statements further mentioned that users can exchange all or part of their reputation points for digital assets in an online market, keep them to maintain their reputation level, or sell it in the future if they think the value of the assets would increase (see Appendix A7 for the full text shown to all treated participants).

We then presented participants with news headlines and, following standard practice [e.g., A2, A3] , asked them about their sharing intentions: “Would you consider posting this news headline on your social media timeline?”. The response options were limited to “no” and “yes”, and the order in which these two options appeared was fully randomized. Although hypothetical, previous research shows that self-reported intentional and actual sharing decisions of news articles are strongly correlated^35^. A detailed description of the questions and workflow is included in the preregistration materials.

### Procedure

First, all participants answer questions about their socio-demographic characteristics, social media use, and various political attitudes and beliefs (see pre-survey questions in Appendix S6). Then each participant is randomly assigned to either the control group (20% of the sample) or one of four treatment conditions (20% of the sample each). The four treatment conditions combine two factors: (1) whether users can only be rewarded for the content they post (Group 3 and 4), or also penalized (Group 1 and 2); and (2) whether the link between their behavior and rewards/penalties is clear (Group 1 and 3) or not (Group 2 and 4). We use simple randomization, not conditional on any observable characteristics of participants.

**Table 1:** Treatment groups.

	Link: Clear^a^	Link: Vague^b^
Rewards and penalties	Group 1	Group 2
Rewards but no penalty	Group 3	Group 4

^a^This condition captures the situation in which the method being used to convert user engagements to reputation points is clearly stated for the participants.

^b^This condition captures the situation in which the method being used to convert user engagements to reputation points is vaguely stated for the participants.

After treatment assignment, all participants in the treatment condition were presented with the following background information about the recent trends in social media platforms:

“[An image of a news headline titled ‘Twitter Fully Incorporates Ethereum Tipping and Wallet Support’.] There have been some recent developments in social media companies to provide an opportunity for their users to gain monetary rewards for the user engagements (number of likes, shares, comments, and video views) they receive. For example, Reddit announced its plan to convert “community points” to digital credits, which users would be able to sell in an online market. When social media platforms such as Facebook, Instagram, TikTok and Twitter will introduce this new feature, users will be able to convert their engagement points to actual money with a one-click button in their profile.

To make these features more concrete, imagine a scenario in which you can earn money as a result of the amount of likes, shares, comments, and video views you receive from your activity on a social media platform (e.g. Facebook or Reddit). Here is what is going to happen in that hypothetical world for you:

1. First, you have to be patient and build a reputation for yourself by posting content and attracting likes, shares, comments, or video views from other users. This may take a few months.
2. After the above warm up period, you start earning “reputation points” from that platform based on the engagements (e.g. likes, shares, comments, or video views) you received. Generally, the more engagement, the more points you receive.
3. Your reputation points will be shown to others in your profile. The more points you have the more trustable and attractive you will appear to other users. Also, the more points you have, the higher the probability that your posts will be shown to other users by the platform’s ranking algorithms.
4. Once you have enough reputation points, there will be an online market where you can sell all or part of them. Like in the stock market, the price for points in that market is variable: it might go up in the future, go down, or stay the same.”

Participants in the Group 1 condition were given the following instructions: “Imagine a scenario in which you have the possibility to post a link to a news article on your social media timeline. In this hypothetical scenario, you also have the opportunity to earn “reputation points” which can be converted into a digital currency (cryptocurrency). More particularly, you will receive one reputation point for every ten likes, shares, comments, or video views you get. This means that the more engagement your post attracts, the more reputation points you will receive, and therefore the more money you can make. However, posting a link to a news article containing misinformation or hate speech, will cost you half a reputation point. On the following pages, you will be shown a number of headlines. Keeping in mind that the popularity and content of your post determine how much money you can make, for each headline, please indicate whether you would consider posting it on your timeline. First, however, answer the question on the next page.”

In the Group 2 condition, the ‘More particularly, you will receive one reputation point for every ten likes, shares, comments, or video views you get.’ sentence was replaced with ‘The number of reputation points you receive depends on the amount of likes, shares, comments or video views you get.’ In the Groups 3 and 4 conditions, the ‘However, posting a link to a news article containing misinformation or hate speech, will cost you half a reputation point.’ sentence from Groups 1 and 2 instructions was removed respectively.

In both conditions, the response options were simply ‘no’ and ‘yes’. Moreover, participants saw the response options listed as either yes/no or no/yes (randomized across participants-that is, an individual participant only ever saw ‘yes’ first or ‘no’ first).

### Manipulation check

After the survey experiment, each participant was presented with the following two questions:

Please answer the following questions based on the scenarios you have just read:

1. Could you be penalized for posting misinformation on social media? (Yes/No)
2. Was it clear exactly how many reputation points you would gain for the engagements you get on social media? (Yes/No)

Following our pre-analysis plan, we do not exclude those who failed the manipulation check from our dataset, but we rerun the analyses results for those who pass the manipulation check as well as a robustness test.

### Dependent variables

The study considers three sets of outcomes linked to the reported willingness to share different kinds of news headlines.

*Fake news:* Participants will be presented with 5 fake news headlines and asked for each: “If you were to see the above article, would you consider posting it on social media?” (Possible responses: yes/no).

*Hyperpartisan news (polarization):* Participants will be presented with 5 true, hyperpartisan news headlines and asked for each: “If you were to see the above article, would you consider posting it on social media?” (Possible responses: yes/no).

*Neutral news:* Participants will be presented with 5 true, non-partisan news headlines and asked for each: “If you were to see the above article, would you consider posting it on social media?”. (Possible responses: yes/no).

Following^27^, the headlines will be randomly selected from the dataset in^28^. Specifically, we will randomly select 5 fake news headlines, 5 hyperpartisan headlines, and 5 neutral headlines from^28^. All pools are balanced in terms of political bias. The news headlines are sequentially presented in random order for each participant.

### Covariates

The following variables are measured in the pre-survey and used as covariates in the OLS regression (see Appendix S6 for full item wording of these survey questions): gender, age, race, party identification, political interest, social media accounts, social media use, social media sharing behavior, investment experience, and knowledge of cryptocurrencies.

### Statistical analysis

Our preregistration specified that following^13^ all analyses would be performed at the level of the individual response (that is, one data point per response per participant; 0 = no, 1 = yes) using linear regression with robust standard errors clustered on participant and headline. Our statistical analysis estimates average treatment effects (ATEs) using OLS as an estimator. Multiple linear regression is a standard way to adjust for covariates in the analysis of experiments [A4]. Formally, we estimate the following OLS model separately for each type of headlines (true, false, and hyperpartisan) and for each relevant comparison (see Fig. 3 and Table 1):

1 $$\begin{aligned} y_{i,j} = \alpha + \beta _1 {\textbf{T}} + \beta _2{{\textbf{X}}_i} + n_{i,j} + h_{i,j} + \epsilon _{i,j} \end{aligned}$$

where *i* indexes the participants, and *j* indexes the news headlines. The independent variable $y_{i,j}$ is whether participant *i* show willingness to share news headline *j*. $\beta _1$ is the effect of treatment, ${\textbf{T}}$, on the willingness of participants to share news headlines. ${{\textbf{X}}_i}$ is a vector of covariates associated with participant *i* (see  “Covariates” section for a full list of covariates). Since our analysis estimates effects on three different outcomes (neutral and hyperpartisan news, and misinformation), we adjusted *p*-values for multiple hypothesis-testing using the Benjamini–Hochberg procedure^36^.

### Supplementary Information

Supplementary Information.

## Data Availability

All data and codes to reproduce the results are available on a Open Science Foundation (OSF) repository.
