# Supplementary material for: Tokenization of social media engagements increases the sharing of false (and other) news but penalization moderates it
Source: Sci Rep. 2023 Aug 22;13:13703. doi: 10.1038/s41598-023-40716-2 (PMC10444751; doi:10.1038/s41598-023-40716-2)
Supplement: Supplementary file 1 — Supplementary Information. [file 41598_2023_40716_MOESM1_ESM.pdf]

S1 Decentralized Social Media: Background Information

S1.1 What is Cryptocurrency?

A cryptocurrency is a digital asset that is designed to work as a currency through a decentralized network of computers in a way that is nearly impossible to counterfeit or double-spend [1]. The technology that secures the transactions and store the history in a digital ledger is called the *blockchain* technology [2]. Therefore, we can define a cryptocurrency as a digital currency that is built on blockchain technology. Bitcoin was the first cryptocurrency released in 2009. Since then many other cryptocurrency have been introduced. For example, Litecoin and Ethereum were launched on October 2011 and August 2015, respectively.

Although initially designed to be a peer-to-peer electronic cash system [3], Bitcoin, and other cryptocurrencies created afterward (a.k.a., alternative coins or altcoins), rapidly gained the reputation of being pure speculative assets [4]. Their prices are mostly idiosyncratic [5], subject to market conditions [6], mainly driven by behavioral factors [7], and are correlated with some major classes of financial assets (e.g. US Dollar index) and uncorrelated with some others (e.g. gold prices) [8].

S1.2 Where does the Monetary Value of Cryptocurrency Come From?

The monetary value of some cryptocurrencies such as bitcoin and etherium comes from the fact that implementing the blockchain technology needs a computing infrastructure, which is costly (e.g. building facilities, electricity bills, and high-performance computers) [9]. The process of maintaining and securing the ledger through the blockchain technology is called *mining* [9]. Other cryptocurrencies earn their value based on a business behind them. For example, a new company can emerge for decentralized money exchange, in which users can send money to anyone in the world without relying on banks, and introduce a new cryptocurrency (i.e. coin) for a blockchain technology that supports its business. The process of converting a physical asset (e.g. a building or land) or a business to a cryptocurrency is called *tokenization* [9].

S1.3 What is NFT and How Does it Create Value?

A new form of tokenization, called non-fungible token (NFT), has recently emerged that tokenizes digital data such photos, videos, and audio. An NFT is a unit of data stored on a blockchain that certifies a digital asset to be unique and therefore not interchangeable, while offering a unique digital certificate of ownership for the NFT [10]. While initial NFTs were all part of the Ethereum (a decentralized open-source blockchain with smart contract functionality), more blockchains are implementing their own version of NFTs [11].

NFTs has been embraced by high-profile musicians, singers, and athletes, as well as digital art creators [10]. Each week, hundreds of millions of dollars of NFTs are being traded on online marketplaces such as OpenSea [3]. Though complex to grasp for many, NFT brings many advantages for digital art market: 1) solves the problem of ownership by storing the digital identity of the owner on a public-facing digital ledger (i.e. blockchain); 2) facilitates the transferring of the rights of use, 3) makes the market transparent by keeping the history of all trades; 4) and facilitates development of a highly-engaged community for digital artists (think it as a membership card).

In summary, NFTs are creating new markets based on new forms of ownership. The value of NFTs, in essence, is coming from the same core dynamics of cryptocurrencies, which is users' shared agreement [12]. In other words, an NFT value comes from the users community one builds around it, and the more this community attracts engagements from users and become part of their everyday life, the more the value increases.

S2 Existing Decentralized Social Media Platforms

Table S.1 shows a list of major existing decentralized social media platforms along with their release year and the major platform they are alternative to them.

No.	Name	Alternative To	Release Year
1	Diaspora	Facebook	2010
2	LBRY	Youtube	2015
3	Minds	Facebook/Twitter	2015
4	Mastodon	Twitter	2016
5	Element	Whatsapp/Slack	2016
6	Dtube	Youtube	2016
7	Steemit	Medium	2016
8	Indorse	Linkedin	2017
9	Emanate	Spotify	2017
10	Peertube	Youtube	2018
11	Peepeth	Twitter	2018
12	Entre	Twitter/Linkedin	2019
13	Subsocial	Any platform	2020

Table S.1 List of the some of the well-know existing decentralized social media platforms.

S3 Recent Plans of Platforms to Financially Reward User Engagements

- Twitter: Tips (https://blog.twitter.com/en_us/topics/product/2021/bringing-tips-to-everyone, accessed August 12, 2022)

[3] https://opensea.io/

- Twitter: Creator monetization for all forms of content (https://twitter.com/elonmusk/status/1589010272341340160?s=20&t=vtBPJ4kqdVoHmfKf2Yj28w, accessed November 8, 2022)
- Medium: Partner Program (https://help.medium.com/hc/en-us/articles/115011694187-Getting-started-with-the-Partner-Program, accessed August 12, 2022)
- Reddit: Community Points (https://www.reddit.com/community-points/, accessed November 8, 2022)
- Reddit: tokenization of *Karma* points (https://cointelegraph.com/news/reddit-to-reportedly-tokenize-karma-points-and-onbo accessed August 12, 2022)

S4 Token Classification Framework

As discussed in the main text, Freni et al (2022) proposed a framework for classification of tokens. Below we provide defintions of the five categories mentioned in the 'Introduction' part of the paper:

1. Technology domain: refers to all technical characteristics of a token, including the level of integration, the blockchain technology, and protocol. The Technology Domain comprises five dimensions: Chain, Permission, Number of Blockchains, Representation Type.
2. Behavior domain: includes the intrinsic characteristics of a token which determines the actions that can be performed with the token. The behaviour domain includes six dimensions: Burnability, Expirability, Spendability, Fungibility, Divisibility, Tradability.
3. Inherent Value domain: refers to the characteristics of a token that define the economic value of the token, how it is originated, and what factors affect its price and fluctuations.
4. Coordination domain: consists the characteristics of a token which enable coordination among the holders of that token. The coordination domain includes four dimensions: Underlying Value, Supply Strategy, Incentive Enablers, Incentive Drivers.
5. Pseudo-archetypes: describes the characteristics of a token "that anticipate a set of token archetypes since they implicitly combine different token characteristics" (Freni et al 2022).

S5 Regression Analysis of Study 1

In study 1, n = 1,022 American individuals from Prolific were presented with five statements about cryptocurrencies of which two were correct and three incorrect which they had to rate as either as 'right' or 'wrong'. In general, the results show that the more participants think they know about cryptocurrency or have more positive opinion about it, the less likely they answer correctly to questions.

Table S.2 reports the results of our first study, which focused on knowledge regarding cryptocurrencies. In the survey, respondents were presented with five statements of which two were correct and three incorrect which they had to rate as either 'right' or 'wrong' . In the first column, the dependent variable is the share of correct answers, whereas in the second it is the number. In both models, the intercept shows the outcome for the reference groups. On average, respondents in the reference groups answered about 46% of the questions correctly, that is about 2.3 questions. We observe that there is little variation in correct response rates. The only significant differences, in a negative direction, are found for older respondents (65+, relative to 18-24 years old) and, interestingly, respondents who reported to be "extremely knowledgeable" about crypto (relative to those reporting to be "not at all knowledgeable"). Based on our questions, both groups have a poorer understanding of crypto than the corresponding reference categories. These results suggest that the understanding of crypto is, on average, mediocre, with little variation across most groups.

Table S.2: *Correlates of understanding of crypto (linear regression models)*

	Correct answers: Percent	Correct answers: Number
(Intercept)	0.46***	2.31***
	(0.11)	(0.57)
genderMale	0.01	0.07
	(0.01)	(0.07)
genderOther	0.09	0.43
	(0.07)	(0.36)
age25-34	0.00	0.01
	(0.02)	(0.12)
age35-44	−0.03	−0.17
	(0.03)	(0.13)
age45-54	−0.03	−0.16
	(0.03)	(0.13)
age55-64	0.00	0.01
	(0.03)	(0.13)
age65+	−0.06*	−0.29*
	(0.03)	(0.14)
raceAmerican Indian or Alaskan Native	−0.10	−0.52
	(0.11)	(0.55)
raceAsian or Asian American	−0.01	−0.03
	(0.06)	(0.30)
raceBlack or African American	−0.05	−0.25
	(0.06)	(0.28)
raceHawaiian or other Pacific Islander	0.15	0.77
	(0.16)	(0.78)
raceHispanic or Latino	−0.04	−0.20
	(0.06)	(0.30)
raceNon-Hispanic White	−0.03	−0.13

Table S.2: *Correlates of understanding of crypto (linear regression models)*

	Correct answers: Percent	Correct answers: Number
	(0.05)	(0.27)
educationCollege graduate	0.04	0.20
	(0.07)	(0.37)
educationHigh school graduate	0.02	0.08
	(0.08)	(0.38)
educationPost-graduate training/Professional school	0.03	0.17
	(0.08)	(0.38)
educationSome college, no 4-year degree	0.01	0.04
	(0.07)	(0.37)
educationTechnical, trade or vocational school	−0.00	−0.00
	(0.08)	(0.40)
ideologySomewhat conservative	0.02	0.09
	(0.03)	(0.13)
ideologySomewhat liberal	0.01	0.03
	(0.02)	(0.10)
ideologyVery conservative	−0.00	−0.02
	(0.03)	(0.17)
ideologyVery liberal	0.03	0.17
	(0.02)	(0.11)
pid_forcedRepublican Party	0.02	0.11
	(0.02)	(0.12)
news>1 hour	−0.01	−0.06
	(0.02)	(0.12)
news 1 hour	0.00	0.01
	(0.02)	(0.12)
news 30 minutes	0.01	0.04
	(0.02)	(0.11)
news10-20 minutes	−0.01	−0.03
	(0.02)	(0.10)
N social media accounts	−0.00	−0.02
	(0.01)	(0.03)
Investment experience	0.00	0.00
	(0.00)	(0.00)
crypto_knowExtremely Knowledgeable	−0.13*	−0.67*
	(0.06)	(0.31)
crypto_knowKnowledgeable	−0.01	−0.05
	(0.02)	(0.12)
crypto_knowSlightly knowledgeable	−0.03	−0.13
	(0.02)	(0.08)
crypto_knowVery Knowledgeable	−0.05	−0.26
	(0.04)	(0.19)
crypto_realAgree	−0.03	−0.15
	(0.04)	(0.22)
crypto_realDisagree	−0.03	−0.14
	(0.04)	(0.21)

Table S.2: *Correlates of understanding of crypto (linear regression models)*

	Correct answers: Percent	Correct answers: Number
crypto_realNeither agree/disagree	0.01	0.05
	(0.04)	(0.21)
crypto_realStrongly agree	−0.01	−0.05
	(0.05)	(0.26)
crypto_justanotherAgree	0.01	0.05
	(0.04)	(0.19)
crypto_justanotherDisagree	0.02	0.12
	(0.04)	(0.19)
crypto_justanotherNeither agree/disagree	−0.01	−0.03
	(0.04)	(0.19)
crypto_justanotherStrongly agree	−0.06	−0.28
	(0.04)	(0.22)
crypto_scamAgree	0.05	0.25
	(0.03)	(0.16)
crypto_scamDisagree	0.03	0.13
	(0.02)	(0.12)
crypto_scamNeither agree/disagree	0.01	0.05
	(0.03)	(0.13)
crypto_scamStrongly agree	−0.01	−0.03
	(0.05)	(0.23)
R^2	0.06	0.06
Adj. R^2	0.02	0.02
Num. obs.	1022	1022

$^{***}p < 0.001$; $^{**}p < 0.01$; $^{*}p < 0.05$

S6 Power Analysis

We preregistered a target sample of 1,500 participants based on a power analysis aiming to obtain 0.80 power to detect a small effect size of 0.02 at the standard 0.05 alpha error probability (Fig S.1). We also used a Bonferroni adjustment by dividing the nominal alpha level, 0.05, by the number of models tested, 15 (5 hypotheses, 3 outcomes), yielding an alpha of 0.003. The adjusted alpha level was used in the power analysis. The analysis revealed that we needed to recruit at least 730 participants.

S7 Pre-Survey

1. GENDER: How do you describe yourself?
   (a) Male
   (b) Female
   (c) Other
2. AGE: Which of the following categories includes your current age?
   (a) 17 or younger
   (b) 18 to 24

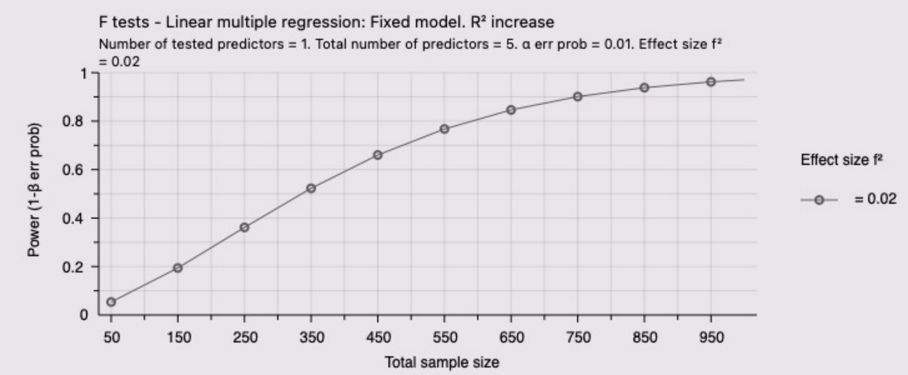

Fig. S.1 Power analysis to aiming to obtain 0.80 power to detect a small effect size of 0.02 at the standard 0.05 alpha error probability. We also used a Bonferroni adjustment by dividing the nominal alpha level, 0.05, by the number of models tested, 15 (5 hypotheses, 3 outcomes), yielding an alpha of 0.003. The adjusted alpha level was used in the power analysis. The analysis revealed that we needed to recruit at least 730 participants.

   (c) 25 to 34
   (d) 35 to 44
   (e) 45 to 54
   (f) 55 to 64
   (g) 65+
3. RACE: How do you describe yourself? (Please check the one option that best describes you)
   (a) American Indian or Alaskan Native
   (b) Hawaiian or other Pacific islander
   (c) Asian or Asian American
   (d) Black or African American
   (e) Hispanic or Latino
   (f) Non-Hispanic White
   (g) Other
4. EDUCATION: What is the highest level of education you have completed?
   (a) None, or grades 1-8
   (b) High school incomplete (grades 9-11)
   (c) High school graduate (grade 12 or GED certificate)
   (d) Technical, trade or vocational school AFTER high school
   (e) Some college, no 4-year degree (includes associate degree)
   (f) College graduate (B.S., B.A., or other 4-year degree)
   (g) Post-graduate training/professional school after college (toward a Master's degree or Ph.D., Law or Medical school)
5. IDEOLOGY: In politics, people sometimes talk about liberal and conservative. In general, would you describe yourself as:
   (a) Very liberal
   (b) Somewhat liberal
   (c) Moderate

   (d) Somewhat conservative
   (e) Very conservative
6. PID: Generally speaking, do you think of yourself as a Democrat, a Republican, an Independent, or something else?
   (a) Strong Democrat
   (b) Weak Democrat
   (c) Lean Democrat
   (d) Independent
   (e) Lean Republican
   (f) Weak Republican
   (g) Strong Republican
7. PID - FORCED: If you absolutely had to choose between only the Democratic and Republican Party, which would you prefer?
   (a) Democratic Party
   (b) Republican Party
8. NEWS: On a typical day, how much time would you say you spend reading the news (either on a mobile phone or a computer?
   (a) Less than 10 minutes
   (b) Between 10-20 minutes
   (c) About 30 minutes
   (d) About 1 hour
   (e) More than 1 hour
9. POLITICAL INTEREST: How interested are you in politics?
   (a) Not at all interested
   (b) Not very interested
   (c) Somewhat interested
   (d) Very interested
   (e) Extremely interested
10. SOCIAL MEDIA ACCOUNT 1: Do you have a social media profile (e.g., Facebook, Twitter, Instagram, Reddit, TikTok)?
   (a) No
   (b) Yes
11. SOCIAL MEDIA ACCOUNT 2: Do you have accounts on any of the following social media sites? Please select all that apply
   (a) Twitter
   (b) Instagram
   (c) Reddit
   (d) TikTok
   (e) None
12. SHARING BEHAVIOR: Would you ever consider sharing something political on social media?
   (a) No
   (b) Yes

13. SOCIAL MEDIA USE: On a typical day, how much time would you say you spend on social media (such as Facebook and Twitter; either on a mobile or a computer)?
   (a) Less than 30 minutes
   (b) 30-60 minutes
   (c) 1-2 hours
   (d) 2-3 hours
   (e) 3+ hours
14. SHARING POST: When deciding whether to share a piece of content on social media, how important is it to you that the content is (Likert scale):
   (a) Surprising
   (b) Politically aligned
   (c) Funny
   (d) Interesting
   (e) Accurate
15. INVESTMENT EXPERIENCE: Do you have any experience in financial trading markets such as stocks, other investments or cryptocurrency (e.g. Bitcoin, Ethereum, NFTs)? Please check all that apply.
   (a) Stocks
   (b) Cryptocurrency
   (c) Other investments
   (d) None
16. KNOWLEDGE OF CRYPTOCURRENCY: How do you evaluate your knowledge of cryptocurrencies (e.g., Bitcoin, Ethereum, NFTs)? (Likert scale)
17. ATTITUDE ON CRYPTOCURRENCY. How much do you agree with the following statements? (Likert scale)
   (a) Cryptocurrencies are real thing with high growth potential in the future
   (b) Cryptocurrencies are just another investment opportunity like the stock market
   (c) Cryptocurrencies are a scam

S8 Treatments

Each participant is randomly assigned to either the control group (20% of the sample) or one of four treatment conditions (20% of the sample each). The four treatment conditions combine two factors: (1) whether users can only be rewarded for the content they post (Group 3 and 4), or also penalized (Group 1 and 2); and (2) whether the link between their behavior and rewards/penalties is clear (Group 1 and 3) or not (Group 2 and 4). We use simple randomization, not conditional on any observable characteristics of participants.

S8.1 Background Information

Participants in the treatment groups will be asked to read the following text, explaining that social media platforms are increasingly considering implementing a system

which provides an opportunity for their users to gain monetary rewards for the engagements that their content attracts.

> "[An image of a news headline titled 'Twitter Fully Incorporates Ethereum Tipping and Wallet Support'.] There have been some recent developments in social media companies to provide an opportunity for their users to gain monetary rewards for the user engagements (number of likes, shares, comments, and video views) they receive. For example, Reddit announced its plan to convert "community points" to digital credits, which users would be able to sell in an online market. When social media platforms such as Facebook, Instagram, TikTok and Twitter will introduce this new feature, users will be able to convert their engagement points to actual money with a one-click button in their profile.
>
> To make these features more concrete, imagine a scenario in which you can earn money as a result of the amount of likes, shares, comments, and video views you receive from your activity on a social media platform (e.g. Facebook or Reddit). Here is what is going to happen in that hypothetical world for you:
>
> 1. First, you have to be patient and build a reputation for yourself by posting content and attracting likes, shares, comments, or video views from other users. This may take a few months.
>
> 2. After the above warm up period, you start earning "reputation points" from that platform based on the engagements (e.g. likes, shares, comments, or video views) you received. Generally, the more engagement, the more points you receive.
>
> 3. Your reputation points will be shown to others in your profile. The more points you have the more trustable and attractive you will appear to other users. Also, the more points you have, the higher the probability that your posts will be shown to other users by the platform's ranking algorithms.
>
> 4. Once you have enough reputation points, there will be an online market where you can sell all or part of them. Like in the stock market, the price for points in that market is variable: it might go up in the future, go down, or stay the same."

S8.2 Four Treatment Groups

Moreover, participants in the treatment groups will asked to read one of following texts:

S8.2.0.1 Group 1:

"Imagine a scenario in which you have the possibility to post a link to a news article on your social media timeline. In this hypothetical scenario, you also have the opportunity to earn "reputation points" which can be converted into a digital currency (cryptocurrency). More particularly, you will receive one reputation point for every ten likes, shares, comments, or video views you get. This means that the more engagement your post attracts, the more reputation points you will receive, and therefore the more money you can make. However, posting a link to a news article containing misinformation or hate speech, will cost you half a reputation point.

On the following pages, you will be shown a number of headlines. Keeping in mind that the popularity and content of your post determine how much money you can make, for each headline, please indicate whether you would consider posting it on your timeline. First, however, answer the question on the next page."

S8.2.0.2 Group 2:

"Imagine a scenario in which you have the possibility to post a news headline on your social media timeline. In this hypothetical scenario, you also have the opportunity to earn "reputation points" which can be converted into a digital currency (cryptocurrency). The number of reputation points you receive depends on the amount of likes, shares, comments or video views you get. This means that the more engagement your post attracts, the more points you will receive, and therefore the more money you can make. However, the exact formula that the platform use to compute your reputation points based on the user engagements you received in unknown. In addition, posting a news headline containing misinformation or hate speech will decrease your reputation points.

On the following pages, you will be shown a number of headlines. Keeping in mind that the popularity and content of your post determine how much money you can make, for each headline, please indicate whether you would consider posting it on your timeline. First, however, answer the question on the next page."

S8.2.0.3 Group 3:

"Imagine a scenario in which you have the possibility to post a link to a news article on your social media timeline. In this hypothetical scenario, you also have the opportunity to earn "reputation points" which can be converted into a digital currency (cryptocurrency). You will receive one reputation point for every ten likes, shares, comments, or video views you get. This means that the more engagement your post attracts, the more points you will receive, and therefore the more money you can make.

On the following pages, you will be shown a number of headlines. Keeping in mind that the popularity of your post determines how much money you can make, for each headline, please indicate whether you would consider posting it on your timeline. First, however, answer the question on the next page."

S8.2.0.4 Group 4:

"Imagine a scenario in which you have the possibility to post a news headline on your social media timeline. In this hypothetical scenario, you also have the opportunity to earn "reputation points", which can be converted into a digital currency (cryptocurrency). The number of reputation points you receive depends on the amount of likes, shares, comments or video views you get. This means that the more engagement your post attracts, the more points you will receive, and therefore the more money you can make. However, the exact formula that the platform use to compute your reputation points based on the user engagements you received in unknown.

On the following pages, you will be shown a number of headlines. Keeping in mind that the popularity of your post determines how much money you can make, for each headline, please indicate whether you would consider posting it on your timeline. First, however, answer the question on the next page."

S9 Debriefing

All participants will be presented with the following debriefing note:

"Thank you for your participation in this research study. Now that you completed or have ended your participation, we will provide you with some additional information about the purposes of this study.

What you should know about this study

The main purpose of this study was for us to observe how likely participants were to share various kinds of news headlines, depending on the rewards and penalties implied by the implementation of crypto technology by social media platforms. The news headlines were real, and were selected to be either false, hyperpartisan, or neutral. It is thus important for you to keep in mind that these were not personally targeted towards you and that they were purposefully misleading and/or hyperpartisan (when they were not neutral).

If you have questions

The main researchers conducting this study are Dr. Meysam Alizadeh (meysam.alizadeh@uzh.ch), Prof. Fabrizio Gilardi (gilardi@ipz.uzh.ch) and Dr. Emma Hoes (hoes@ipz.uzh.ch) (University of Zurich, Department of Political Science). If you have questions, you may contact one of the main researchers. If you have any questions or concerns regarding your rights as a research participant in this study, you may contact the Ethics Committee: Prof. Dr. Lilly Shanahan Universität Zürich Jacobs Center for Productive Youth Development Andreasstrasse 15, P.O. Box 12 CH-8050 Zürich""

S10 Robustness Check

As mentioned in Section 4.1, 30% of participants did not pass the manipulation test. To check for the robusteness of our results, we remove those who failed the manipulation check and report the OLS estimates in Fig S.2.

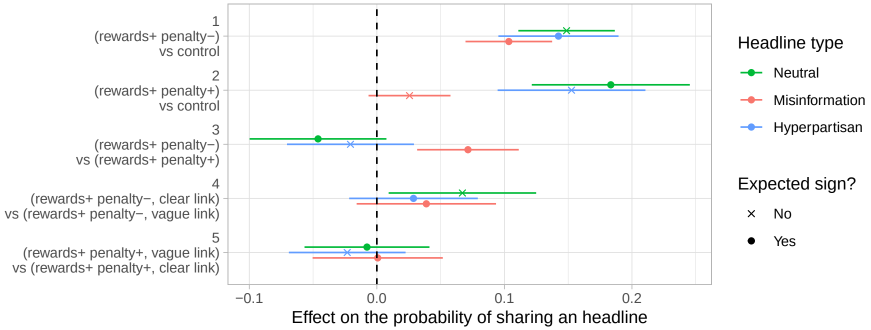

Fig. S.2 Point estimates from OLS regressions with 95% CI for participants who passed the robustness check. Effect of monetary rewards for user engagements and penalties for problematic sharing behavior, as well as of the clarity of the link between rewards/penalties and engagements/behavior, on the willingness to share neutral and hyperpartisan news, and misinformation. Without penalties, rewards increase the willingness to share misinformation by about nine percentage points, compared to the control group. With penalties, the effect decreases to about four percentage points. The clarity of the link has no effect.

Appendix References

[A1] Joseph Bonneau, Andrew Miller, Jeremy Clark, Arvind Narayanan, Joshua A Kroll, and Edward W Felten. Sok: Research perspectives and

challenges for bitcoin and cryptocurrencies. In *2015 IEEE symposium on security and privacy*, pages 104–121. IEEE, 2015.

[A2] Dylan Yaga, Peter Mell, Nik Roby, and Karen Scarfone. Blockchain technology overview. *arXiv preprint arXiv:1906.11078*, 2019.

[A3] Satoshi Nakamoto. Bitcoin: A peer-to-peer electronic cash system. *Decentralized Business Review*, page 21260, 2008.

[A4] David Yermack. Is bitcoin a real currency? an economic appraisal. In *Handbook of digital currency*, pages 31–43. Elsevier, 2015.

[A5] Helder Sebastião and Pedro Godinho. Forecasting and trading cryptocurrencies with machine learning under changing market conditions. *Financial Innovation*, 7(1):1–30, 2021.

[A6] Fan Fang, Carmine Ventre, Michail Basios, Leslie Kanthan, David Martinez-Rego, Fan Wu, and Lingbo Li. Cryptocurrency trading: a comprehensive survey. *Financial Innovation*, 8(1):1–59, 2022.

[A7] Mehmet Balcilar, Elie Bouri, Rangan Gupta, and David Roubaud. Can volume predict bitcoin returns and volatility? a quantiles-based approach. *Economic Modelling*, 64:74–81, 2017.

[A8] Yechen Zhu, David Dickinson, and Jianjun Li. Analysis on the influence factors of bitcoin's price based on vec model. *Financial Innovation*, 3(1):1–13, 2017.

[A9] Arvind Narayanan, Joseph Bonneau, Edward Felten, Andrew Miller, and Steven Goldfeder. *Bitcoin and cryptocurrency technologies: a comprehensive introduction*. Princeton University Press, 2016.

[A10] Matthieu Nadini, Laura Alessandretti, Flavio Di Giacinto, Mauro Martino, Luca Maria Aiello, and Andrea Baronchelli. Mapping the nft revolution: market trends, trade networks, and visual features. *Scientific reports*, 11(1):1–11, 2021.

[A11] The Writer's Lounge. Choosing the right blockchain for your nft. Technical report, Medium, 2020.

[A12] Steve Kaczynski and Duke Kominers. How nfts create value. Technical report, Harvard Business Review, 2021.
